# Single-cell Sequencing and Machine Learning Identify Amino Acid Metabolism-related Biomarkers and Regulatory Mechanisms in Diabetic Foot Ulcers

**DOI:** 10.2174/0118715303451779260511203500

**Published:** 2026-05-21

**Authors:** Wenting Wang, Zhengguo Xia, Yin Wang, Fan Wang, Feng Han

**Affiliations:** 1 Wound Repair & Plastic and Anesthetic Surgery, the First Affiliated Hospital of Anhui Medical University, North District, Hefei City, Anhui Province 230022, China

**Keywords:** Single-cell sequencing, machine learning, amino acid metabolism, diabetic foot ulcers, cell-cell communication, keratinocyte subsets

## Abstract

**Introduction:**

Diabetic foot ulcer (DFU) is a serious complication of diabetes with poor healing and high mortality, and effective diagnostic and treatment strategies are still insufficient.

**Methods:**

Single-cell RNA sequencing dataset GSE165816 was processed for quality control, normalization, dimensionality reduction, clustering, and annotation. Keratinocyte subsets were analyzed using pseudotime trajectory inference, cell-cycle profiling, and assessment of transcription factor activity. Cell-cell communication was evaluated through cadherin signaling analysis. Differential expression and enrichment analyses were performed to identify subgroup-specific functional pathways. A bulk RNA sequencing dataset (GSE134431) was utilized to screen amino acid metabolism-related genes using machine learning approaches, followed by external dataset validation, ROC analysis, molecular docking, and qPCR.

**Results:**

Single-cell RNA sequencing identified 13 cell types, among which keratinocytes showed significant heterogeneity. Four keratinocyte subsets were defined, among which Kera1 exhibited strong stemness, initiated differentiation toward Kera3, and showed distinct functional states across groups. Cell-cell communication analysis revealed enhanced cadherin signaling in DFU non-healing samples, particularly driven by Kera1 autocrine CDH1-CDH1 interactions. Transcription factor analysis highlighted CEBPA and KLF5 as key regulators of Kera1. Machine learning integrated with bulk RNA sequencing identified five amino acid metabolism-related genes (RPL13, ODC1, RPL22L1, GATM, GLUL) with diagnostic value. qPCR further confirmed the dysregulated expression of these genes in clinical samples. Molecular docking suggested ODC1 as a potential therapeutic target for Eflornithine.

**Discussion:**

The identification of Kera1-driven cadherin signaling and five key metabolic biomarkers offers a mechanism-based framework for clinical diagnosis and targeted therapy, potentially shifting DFU management toward more precise, molecular-level interventions.

**Conclusion:**

Keratinocyte stemness and subtype-specific differentiation contribute to altered cadherin signaling in DFU, while key amino acid metabolism-related genes serve as diagnostic biomarkers and therapeutic targets.

## 1 INTRODUCTION

Diabetic foot ulcers (DFUs) represent one of the most common and serious chronic complications of diabetes [1]. DFUs are characterized by persistent destruction of the skin and underlying tissues, which may result in infection, gangrene, and even amputation in severe cases [2, 3]. The development of DFUs is closely associated with multiple factors, including microangiopathy and neuropathy induced by chronic hyperglycemia, insufficient blood supply resulting from peripheral arterial disease, trauma, infection, and impaired immune function [4, 5]. Epidemiological studies indicate that approximately 15-25% of people with diabetes worldwide will develop DFUs during their lifetime [6]. These conditions markedly increase morbidity and mortality, while severely impairing quality of life and increasing the overall healthcare burden [7]. Despite recent advances in understanding the pathogenesis, diagnosis, and treatment of DFUs, current therapeutic strategies remain primarily focused on glycemic control, infection prevention, restoration of blood supply, and wound repair [8, 9]. However, overall efficacy is limited, and patient prognosis is generally poor, with recurrence and amputation risks remaining high. Existing research has primarily focused on clinical interventions and the assessment of conventional indicators, whereas the molecular mechanisms underlying DFU development and progression, as well as their specific biomarkers, remain insufficiently understood. Therefore, a deeper understanding of the molecular mechanisms underlying DFUs, along with the identification of novel diagnostic markers, therapeutic targets, and prognostic indicators, is essential for improving patient outcomes and reducing the disease burden.

Amino acid metabolism plays a central role in maintaining physiological homeostasis, serving not only as a source of substrates for protein synthesis but also as a key regulator of numerous physiological processes, including energy metabolism, redox balance, and signal transduction [10, 11]. In addition, amino acid metabolites play critical roles in modulating inflammatory responses and immune function. For example, tryptophan metabolism regulates oxidative stress and apoptosis, whereas glutamine and arginine contribute to tissue repair and angiogenesis [12], while glutamine and arginine play key roles in tissue repair and angiogenesis [13]. Recent studies have demonstrated that dysregulation of amino acid metabolism is closely associated with diabetes. Elevated levels of branched-chain amino acids have been strongly associated with insulin resistance and an increased risk of type 2 diabetes, whereas reduced levels of specific amino acids are linked to the development of chronic diabetic complications [14, 15]. DFUs represent one of the most severe complications of diabetes. The pathophysiological progression of DFUs involves persistent hyperglycemia, neuropathy, vascular dysfunction, and immune imbalance [16]. These wounds exhibit impaired healing and increased susceptibility to infection, with metabolic abnormalities playing a critical role in this process. Dysregulation of amino acid metabolism can impair energy metabolism and exacerbate local tissue damage by disrupting inflammatory responses and vascular repair, thereby delaying ulcer healing [17, 18]. Therefore, advancing the understanding of DFU at the molecular and cellular levels is essential for shifting from phenotype-based management toward mechanism-based disease stratification, enabling the development of precise diagnostic markers and targeted therapeutic strategies.

Keratinocytes are highly metabolically active cells that rely on amino acid metabolism to support proliferation, differentiation, and stress adaptation during wound repair. Increasing evidence indicates that metabolic dysregulation plays a critical role in the pathogenesis of DFU [19, 20]. However, the cell-type-specific alterations and regulatory mechanisms of amino acid metabolism, particularly at the single-cell level, remain poorly understood. This knowledge gap limits a precise understanding of cellular heterogeneity and its contribution to impaired wound healing [21]. Therefore, this study integrated single-cell transcriptomics and machine learning approaches to identify amino acid metabolism-related keratinocyte subpopulations systematically, characterize key regulatory genes, and evaluate their potential diagnostic value in DFU, thereby providing mechanistic insights and potential therapeutic targets for precision intervention (Fig. **1**).

## 2 MATERIALS AND METHODS

### 2.1 Data Acquisition

Single-cell RNA sequencing (scRNA-seq) data of DFU tissues (GSE165816) and bulk transcriptome datasets (GSE199939, GSE134431, and GSE7014) were retrieved from the Gene Expression Omnibus (GEO, https://www.ncbi.nlm.nih.gov/geo/) (Table **1**). Among them, the scRNA-seq dataset GSE165816 was generated using the GPL24676 platform and included 7 non-healing DFU patients (DFU_NH) and 15 healthy control samples (H). A total of 374 amino acid metabolism-related genes (AAMRGs) were obtained from the Molecular Signatures Database (MSigDB, https://www.gsea-msigdb.org/gsea/msigdb).

### 2.2 Study Design

This was a retrospective, multi-dataset bioinformatic analysis that integrated publicly available single-cell and bulk transcriptome data. The study design included secondary analysis of scRNA-seq data for cell type identification and characterization of keratinocyte subpopulations, differential expression analysis of bulk RNA-seq data for biomarker discovery, and external validation using independent cohorts. Machine learning algorithms were applied for feature selection, followed by molecular docking to explore potential therapeutic targets. Experimental validation of key genes was performed using qPCR on clinical samples.

### 2.3 Single-cell RNA Sequencing Data Processing and Analysis

Quality control of the scRNA-seq dataset GSE165816 was performed using Seurat (v 5.10) in R. Cells were retained based on the following criteria: min.cells = 3, min.features = 200, nFeature RNA < 7,500, nCount RNA < 40,000, and percent.mt < 20%. Normalization of the filtered data was conducted using the NormalizeData function in the R package Seurat (v 5.1.0). Subsequently, highly variable genes were identified with the FindVariableFeatures function, and the ScaleData function was applied to normalize the mean to zero and the variance to one, thereby eliminating scale differences across genes. To reduce the technical variation and batch effects across samples, data integration was performed using the Harmony algorithm *via* the RunHarmony function.

### 2.4 Dimensionality Reduction, Clustering, and Cell Type Annotation in Single-cell Transcriptomic Analysis

Dimensionality reduction of sequencing data using principal component analysis (PCA), and the results were visualized using t-distributed stochastic neighbor embedding (t-SNE) and uniform manifold approximation and projection (UMAP). Cell clustering was conducted using the FindNeighbors and FindClusters functions. In addition, DEGs within each cluster were determined with the FindAllMarkers function, which compares each cluster with all others to identify significantly upregulated or downregulated genes. Finally, cell type annotation was determined by previously published canonical marker genes.

### 2.5 Keratinocyte Re-Clustering And Identification of Key Subpopulations

Keratinocytes represent the predominant cell type in the epidermis and play a pivotal role in the pathogenesis and healing of DFU. The extracted keratinocyte subset was subjected to dimensionality reduction, reclustering, and re-annotation to obtain refined subpopulations. The cellRatioPlot function was applied to quantify the relative proportions of keratinocyte subtypes across different groups. To evaluate amino acid metabolism activity, AUCell was applied to calculate gene set activity scores based on the predefined AAMRG gene set from MSigDB. The resulting AUC scores were incorporated into the single-cell object and compared across keratinocyte subtypes. The median AUC score of each subtype was used to quantify amino acid metabolic activity, and violin plots were generated for visualization. Subpopulations exhibiting relatively high AUC values were defined as potential key subsets involved in DFU.

### 2.6 Analysis of Keratinocyte Subtype Differentiation Trajectories, Intercellular Communication, and Transcription Factor Regulation

Differentiation trajectories of keratinocyte subtypes were inferred using three complementary algorithms, including CytoTRACE, Slingshot, and Monocle, to provide an in-depth understanding of their developmental progression. To further characterize intercellular signaling, the R package CellChat (v 2.1.2) was employed for systematic analysis and visualization of cell-cell communication networks among key cell populations.

### 2.7 Transcription Factor Regulatory Network Analysis

Transcription factor regulatory activity was predicted using Single-Cell Regulatory Network Inference and Clustering (SCENIC, https://scenic.aertslab.org/). TF activity differences among keratinocyte subtypes were assessed, and the top five transcription factors with the highest regulatory activity in each subtype were visualized.

### 2.8 Intergroup Differences and Enrichment Analysis of Key Keratinocyte Subtypes

Differential expression analysis between keratinocyte subtypes was performed using the RunDEtest function in the R package SCP (v 0.5.6). Differentially expressed genes (DEGs) were defined as those with |avg_log_2_FC| > 0.25 and adjusted *P*-value < 0.05, and volcano plots were generated for visualization. Functional enrichment analysis of identified DEGs was subsequently conducted based on gene sets retrieved from the Gene Ontology (GO) database (http://geneontology.org/), to reveal the biological processes associated with the distinct keratinocyte subtypes.

### 2.9 Identification and Enrichment Analysis of Bulk_DEGs

Differential expression analysis of the bulk dataset GSE134431 was performed using the R package limma (v 3.60.4). Genes were considered differentially expressed (hereafter referred to as bulk_DEGs) if they met the thresholds of P-value < 0.05 and |log_2_ fold change (FC)| > 0.5. The identified Bulk_DEGs were subsequently classified into six expression modules (C1-C6) using the fuzzy clustering algorithm mfuzz. Gene Ontology-Biological Process (GO-BP) and Kyoto Encyclopedia of Genes and Genomes (KEGG) pathway enrichment analyses were conducted for each module to explore their functional characteristics.

### 2.10 Machine Learning-based Feature Selection and Diagnostic Evaluation

The intersection of AAMRGs, bulk_DEGs, and Kera1_DEGs (DEGs from potential key keratinocyte subset Kera1) was used to identify disease-associated amino acid metabolism genes. Feature selection was performed using three machine learning algorithms, including least absolute shrinkage and selection operator (Lasso), support vector machine (SVM), and extreme gradient boosting (XGBoost). The overlapping genes of the three algorithms were defined as key candidate genes. Their diagnostic performance was evaluated by constructing receiver operating characteristic (ROC) curves using the pROC package. The training dataset was GSE134431, and validation was performed using GSE199939 and GSE7014.

### 2.11 Prediction and Molecular Docking of Potential Drugs Targeting Key Genes

Potential drugs associated with the identified key genes were predicted using the Drug-Gene Interaction database (DGIdb; https://www.dgidb.org/). The three-dimensional (3D) structures of the corresponding proteins were obtained from the Protein Data Bank (PDB; https://www.rcsb.org/), whereas the 3D structures of candidate drugs were retrieved from PubChem (https://pubchem.ncbi.nlm.nih.gov/). Molecular docking analyses were performed using CB-Dock2 (https://cadd.labshare.cn/cb-dock2/php/index.php) to evaluate the binding affinity between key gene-encoded proteins and their potential drugs. Finally, PyMOL (https://pymol.org/) was applied to visualize the interactions between specific proteins and the docked drug molecules.

### 2.12 Quantitative Polymerase Chain Reaction (qPCR) Analysis

Total RNA was extracted from tissue samples using Trizol reagent (Tiangen, China), followed by chloroform separation and isopropanol precipitation. RNA concentration and purity were measured using a SYNERGY/HTX microplate reader (BioTek, USA). Genomic DNA was removed using the gDNA Eraser Mix (Vazyme, China), and cDNA was synthesized using the HiScript III RT SuperMix for qPCR (Vazyme, China). Real-time PCR was performed using SYBR Green Master Mix (Vazyme, China) on a BIO-RAD CFX384 system (Bio-Rad, USA) with specific primers for key genes.

### 2.13 Statistical Analysis

Statistical analyses were conducted using R software (v 4.4.1), and network visualizations were generated using Cytoscape (v 3.9.1). The Wilcoxon test was used for detecting differences between two groups, and a *P* < 0.05 was considered a significant difference unless specified. For comparison of continuous variables between groups, we employed the Student’s *t*-test for normally distributed data, and the Mann-Whitney U-test (Wilcoxon test) for non-normally distributed data. A two-tailed *P*-value < 0.05 was considered statistically significant unless otherwise stated.

## 3 RESULTS

### 3.1 Single-cell Transcriptome Analysis and Cell Type Annotation

Quality control of the single-cell RNA sequencing dataset GSE165816 yielded a total of 71,376 high-quality cells (Fig. **2A**). After normalization, highly variable genes were identified, and the top 2,000 variable genes were selected for subsequent analyses (Supplementary Figure **1A**). Principal component analysis (PCA) was performed, and based on the elbow plot, 39 principal components were retained for downstream processing (Supplementary Figure **1B**). Following batch effect correction, cells from different samples exhibited a well-integrated distribution (Fig. **2B**). A clustering dendrogram illustrated the hierarchical organization of cell populations across multiple resolutions (Supplementary Figure **1C**). At a resolution of 0.3, a total of 23 clusters were identified (Fig. **2C**). Cluster annotation, guided by previously reported marker genes, classified these clusters into 13 distinct cell types, including smooth muscle cells (SMCs), fibroblasts (Fibro), vascular endothelial cells (VasEndo), T lymphocytes (T-Lympho), and keratinocytes (Kera) (Figs. **2D** and **2E**). Doublets were excluded before subsequent analyses. A heatmap displaying the top three marker genes for each cluster further confirmed cell-type specificity (Fig. **2F**). Cell composition analysis revealed that Fibro, SMCs, and Kera accounted for a substantial proportion of cells in both groups. Notably, Fibros were more abundant in the H group compared with the DFU_NH group, whereas MPs and T-Lympho were enriched in the DFU_NH group (Figs. **2G** and **2H**).

### 3.2 Identification and Functional Characterization of Kera Cell Subsets

Keras has been reported to play a pivotal role in wound healing. To further delineate their heterogeneity, Kera transcriptomes were normalized, and the top 2,000 highly variable genes were retained for downstream analyses (Supplementary Figure **1D**). Principal component analysis identified 34 components based on the elbow plot, which were subsequently used for dimensionality reduction (Supplementary Figure **1E**). After batch effect correction, Keras from different samples displayed a well-integrated distribution (Fig. **3A**). Clustering at a resolution of 0.02 identified five Kera subsets comprising 14,786 cells (Supplementary Figure **1F**, Fig. **3B**). One subset (Cluster 3), likely representing doublets, was excluded, leaving four keratinocyte subtypes designated as Kera1, Kera2, Kera3, and Kera4 (Fig. **3C**). Cell composition analysis revealed that Kera1 accounted for a substantial proportion of cells in both the H and DFU_NH groups. Most Kera1 cells were enriched in the G1 and S phases of the cell cycle, suggesting an active proliferative and DNA synthesis state (Figs. **3D** and **3F**). Functional scoring demonstrated that Kera1, Kera3, and Kera4 exhibited higher Ro/e scores in the H group, whereas Kera2 showed elevated Ro/e scores in the DFU_NH group (Fig. **3G**). The top five marker genes for each Kera subtype were visualized in a heatmap, highlighting distinct transcriptional signatures across clusters (Fig. **3H**). Gene set activity analysis using AUCell indicated that Kera1 displayed the highest AUC score among all subtypes (Figs. **3I** and **3J**), with significant differences observed between the H and DFU_NH groups (Fig. **3K**). These findings indicate that Kera1 represents a key keratinocyte subpopulation with elevated amino acid metabolism activity, suggesting its potential role as a cellular mediator linking metabolic dysregulation to impaired wound healing in DFU.

### 3.3 Kera1 Displays Stemness and Initiates Differentiation Toward Kera3

CytoTRACE analysis demonstrated that Kera1 exhibited the highest score, suggesting that this subpopulation displayed the strongest stem-like characteristics and represented the least differentiated state (Fig. **4A**). Genes positively correlated with CytoTRACE scores, including DMKN and LGALS7B, were predominantly expressed in Kera1, whereas negatively correlated genes, such as KRT14 and KRT5, were enriched in subsets with higher differentiation potential, including Kera2 and Kera3 (Fig. **4B**). Pseudotime trajectory analysis revealed a developmental progression originating from Kera1, transitioning through Kera4 and Kera2, and ultimately converging toward Kera3 (Fig. **4C**). Notably, Kera3 was more prominent in the H group, as shown by both group-specific distributions and density-based visualization along the trajectory (Figs. **4D**-**4F**).

### 3.4 Enhanced CDH Signaling Highlights Enhanced Kera1 Interaction in DFU_NH

Cell-cell communication analysis revealed that, compared with the H group, Kera1 in the DFU_NH group exhibited markedly stronger interactions with fibroblasts, followed by smooth muscle cells (Fig. **5A**). A comparison of global signaling activity across cell types revealed that the cadherin (CDH) signaling pathway was upregulated in the DFU_NH group but downregulated in the H group (Fig. **5B**). Heatmaps depicting functional roles of each cell type within the CDH signaling network illustrated distinct patterns between groups (Figs. **5C** and **5D**). In the H group, melanocytes acted as the predominant senders and receivers of CDH signaling. In contrast, in the DFU_NH group, Kera1 emerged as an additional major sender and receiver, alongside melanocytes. Cell-cell communication probability matrices further demonstrated that melanocyte-melanocyte interactions dominated in the H group (Figs. **5E**-**5G**). However, in the DFU_NH group, both melanocyte-melanocyte and Kera1-Kera1 interactions were significantly enhanced (Figs. **5E**-**5H**). Ligand-receptor pair analysis provided mechanistic insights, showing that Kera1 in the DFU_NH group displayed markedly elevated autocrine signaling mediated by the CDH1-CDH1 pair. Moreover, CDH1-CDH1 interactions between melanocytes and Kera1 were also substantially stronger in the DFU_NH group compared with the H group (Figs. **5I** and **5J**).

### 3.5 Key Transcription Factors and Functional Enrichment of Kera1 in DFU

Activity analysis revealed that CEBPA(+), ZNF467(+), and KLF5(+) displayed relatively high AUC scores in Kera1, with CEBPA(+) and KLF5(+) showing the most pronounced activity (Figs **6A** and **6B**). Further examination of TF expression confirmed that CEBPA(+) and KLF5(+) were significantly upregulated in Kera1 compared with the other three subtypes, suggesting their pivotal role in regulating Kera1 identity (Fig. **6C**). Differential expression analysis identified the top five upregulated and downregulated genes in Kera1 from both the H and DFU_NH groups (Fig. **6D**). Functional enrichment revealed that Kera1 in the H group was mainly associated with apoptotic processes, multivesicular body formation, actin regulation, and oxidative functions. Kera1 in the DFU_NH group was also enriched in these processes but exhibited additional enrichment in stress response and detoxification pathways (Fig. **6E**). GSEA further highlighted distinct functional programs. In the H group, Kera1 was significantly enriched in the sphingolipid metabolic process, driven by key genes such as SMPD1, SPTSSB, and ELOVL1, indicating enhanced lipid metabolic activity. By contrast, Kera1 in the DFU_NH group showed significant enrichment in the positive regulation of peptidase activity, with core driver genes including TNFSF10, CTSL, SERPINB3, and S100A8, reflecting a role in inflammation-mediated proteolysis and extracellular matrix remodeling (Figs. **6F** and **6G**).

### 3.6 Screening of Candidate Genes Related to Amino Acid Metabolism

From the GSE134431 dataset, a total of 2,727 DEGs were identified, including 1,374 upregulated and 1,353 downregulated genes, with the top ten upregulated genes highlighted (Fig. **7A**). These DEGs were classified into six expression modules (C1-C6). Enrichment analysis revealed that C1 and C6 modules were predominantly expressed in tumor samples, whereas C2, C3, C4, and C5 modules were enriched in normal samples (Fig. **7B** and **7C**). To further refine disease-relevant genes, Kera1_DEGs, bulk_DEGs, and AAMRGs were intersected, yielding 22 disease-related AAMRGs (Fig. **7D**). Subsequent feature selection was performed using multiple machine learning algorithms. Lasso regression identified 5 candidate genes (Fig. **7E** and **7F**). SVM analysis indicated optimal model performance with 14 genes (Figs. **7G** and **7H**). XGBoost selected 8 candidate genes (Fig. **7I**). The intersection of genes identified by the three approaches resulted in 5 robust candidate genes: RPL13, ODC1, RPL22L1, GATM, and GLUL (Fig. **7J**). This result further supports the presence of amino acid metabolism dysregulation in DFU and provides molecular evidence consistent with the metabolic alterations observed in keratinocyte subpopulations.

### 3.7 Diagnostic Performance of Key Genes and Identification of Potential Therapeutic Targets in DFU

ROC curves were generated for the training dataset GSE134431 and the external validation datasets GSE199939 and GSE7014. The results demonstrated that RPL13, ODC1, RPL22L1, GATM, and GLUL consistently achieved an AUC value above 0.7 across all datasets, indicating their strong diagnostic potential for DFU (Figs. **8A**-**8F**; Supplementary Figures **2A-2L**). These genes were therefore selected as key biomarkers for subsequent analyses. Predicted drug-gene interactions were further examined, and a drug-gene network was constructed (Fig. **8G**). Among the five genes, RPL13, ODC1, GATM, and GLUL were associated with potential therapeutic agents. Two drugs with the highest interaction scores, Eflornithine hydrochloride and APA, were prioritized. Literature review confirmed that Eflornithine hydrochloride is the salt form of Eflornithine, which is an orally active ODC1 inhibitor already applied in the prevention of relapse in high-risk neuroblastoma. As shown in Table **2**, ODC1 exhibited a binding affinity of -5.2 kcal/mol with Eflornithine. Molecular docking revealed that Eflornithine formed stable interactions with ODC1 at amino acid residues ARG-402, ALA-39, ASP-38, ASN-319, and GLN-116 (Fig. **8H**). These findings suggest that Eflornithine may regulate protein function through hydrogen bonding with specific amino acid residues, thereby influencing gene regulatory networks and disease progression.

### 3.8 Differential Expression of Key Genes in DFU Patients and Controls

Expression analysis revealed that RPL13 and GATM were significantly higher in normal controls than in DFU patients, whereas ODC1 and RPL22L1 were markedly elevated in the DFU group (Figs. **9A** and **9B**). qPCR validation further indicated that RPL13 and GATM exhibited no significant differences between groups, while ODC1, RPL22L1, and GLUL were consistently overexpressed in DFU patients (Figs. **9C**-**9G**).

## 4 DISCUSSION

DFU is a common and serious complication of diabetes, and its development and progression are closely related to metabolic abnormalities [22]. Amino acid metabolism is not only involved in energy metabolism and protein synthesis but also regulates oxidative stress and inflammatory responses. Disruptions in amino acid metabolic pathways may exacerbate tissue damage and delay wound healing [23]. Therefore, it plays a crucial role in the development and progression of DFU. In our study, single-cell transcriptome analysis revealed marked transcriptional differences between immune cells and keratinocytes in DFU tissues, with significant downregulation of amino acid metabolism-related pathways. Further machine learning algorithms identified several key amino acid metabolism-related genes that were closely associated with disease severity and prognosis. qPCR validation confirmed the dysregulated expression of these genes in DFU tissues. Collectively, these findings indicate that dysregulated amino acid metabolism may accelerate DFU progression by modulating immune responses and epidermal repair, thereby contributing to keratinocyte dysfunction and impaired wound healing.

Single-cell sequencing identified 13 cell types, including keratinocytes (Kera), which were further divided into four subpopulations. Kera1 exhibits high stemness and differentiates into Kera3, with varying proportions and functions among different subpopulations. Keratinocytes, as the primary cell component of the epidermis, play a central role in maintaining the skin barrier, protecting against external damage, and promoting wound repair. Their normal migration, proliferation, and differentiation are crucial for wound healing [24]. However, in diabetic patients, high glucose levels, oxidative stress, and chronic inflammation can impair keratinocyte function, leading to decreased skin repair capacity [25, 26]. In diabetic conditions, keratinocytes exhibit impaired barrier function and delayed wound healing, with decreased migration, adhesion, and proliferation [27]. Furthermore, imbalanced regulation of secreted signaling molecules further disrupts intercellular interactions and impairs wound repair. The study also found that circRNA-080968 was significantly upregulated in DFU tissues, primarily localized in the cytoplasm. Its overexpression inhibited keratinocyte migration and promoted proliferation [28]. Mechanistically, circRNA-080968 binds to miR-326 and miR-766-3p, accelerating their degradation, thereby upregulating the expression of genes regulating cell adhesion and proliferation, leading to keratinocyte dysfunction and further exacerbating DFU pathology. Furthermore, previous studies have demonstrated that multiple molecular pathways and epigenetic regulation are involved in regulating keratinocyte activity, and their abnormal expression can significantly affect wound healing [29]. In summary, keratinocyte dysfunction plays a central role in the development and progression of DFU, and its molecular regulatory mechanisms provide potential targets for future intervention and treatment.

Our study found that in the DFU_NH group, Kera1 cells showed significantly enhanced autocrine secretion and enhanced autocrine signaling, and increased interactions with fibroblasts and melanocytes *via* CDH-mediated signaling. Furthermore, the activities of the key Kera1 transcription factors CEBPA and KLF5 were elevated. Functional enrichment analysis revealed a significant enrichment in stress response, detoxification, and inflammation-mediated proteolysis, whereas in the H group, functional enrichment was primarily associated with sphingolipid metabolism and cytoskeletal regulation. Although research on proteolysis and sphingolipid metabolism in DFU remains limited, in the broader context of diabetes research, suggesting that these metabolic pathways may also contribute to the pathological process of DFU. Proteolysis, the enzymatic breakdown of proteins into amino acids, maintains cellular metabolism and protein turnover [30]. Proteolysis and protein peroxidation were significantly enhanced in the cerebral cortex and hippocampus of type 2 diabetic rats. Enalapril treatment reduced proteolytic activity and alleviated neuronal damage [31]. Sphingolipid metabolism is a key pathway regulating cell membrane structure and signal transduction, maintaining cellular homeostasis [32]. Studies have shown that patients with diabetic peripheral neuropathy have abnormalities in multiple metabolites in serum and urine, including sphingolipid-related metabolites such as sphingosine and sphingine [33]. Furthermore, abnormal expression of sphingolipid metabolism-related genes S1PR1 and SELL in diabetic nephropathy is closely associated with cytokine receptor interactions and immune cell changes [34].

DEGs identified from the GSE134431 dataset were divided into six modules. The results showed that C1 and C6 were highly expressed in tumor samples and enriched in protein folding, mitosis, immunity, and AGE-RAGE signaling pathways. C2 to C5, on the other hand, were highly expressed in normal samples and primarily involved in epidermal/keratinogenesis, immune regulation, and basal metabolism. Mitosis is a form of nuclear division that accurately transfers genetic material to daughter cells through chromosome replication and equal distribution, ensuring cell proliferation and tissue growth [35]. In DFU wounds, impaired mitotic activity contributes to delayed healing, whereas fibroblast growth factors (FGFs) can restore cell proliferation and angiogenesis [36]. The AGE-RAGE signaling pathway, activated by the binding of advanced glycation end products (AGEs) to their receptor RAGE, regulates inflammation, oxidative stress, and cellular function [37]. Studies have shown that cinnamaldehyde can promote macrophage M2 polarization, enhance fibroblast activation and angiogenesis, and accelerate wound healing by inhibiting AGE-RAGE signaling in DFU wounds [38]. Furthermore, significant activation of AGE-RAGE signaling in DFU wounds is associated with cellular senescence and the SASP, the regulation of which can influence inflammation levels and wound repair rates [39]. Taken together, these results reveal the crucial role of mitosis and AGE-RAGE signaling in the pathological process of DFU, providing scientific evidence for the study of related mechanisms.

Through multiple machine learning methods, we identified five key genes: RPL13, ODC1, RPL22L1, GATM, and GLUL. Their diagnostic performance was validated across independent datasets. Among them, ODC1, RPL22L1, and GLUL were significantly overexpressed in patients with DFU, and ODC1 showed potential interactions with the ODC inhibitor eflornithine hydrochloride. ODC1 encodes ornithine decarboxylase (ODC), the rate-limiting enzyme in polyamine biosynthesis, which regulates cell proliferation and differentiation [40]. Its abnormal activation is closely associated with oxidative stress and metabolic disorders, playing a crucial role in the development and progression of diabetes [41]. Studies have shown that impaired glycolysis in diabetic podocytes leads to enhanced ODC1-mediated ornithine catabolism, which in turn promotes mTOR signaling and induces podocyte cytoskeletal remodeling [42]. Eflornithine hydrochloride is an irreversible ODC inhibitor that primarily targets polyamine metabolism. Studies have shown that increased ODC activity and impaired polyamine metabolism are common in diabetic patients and are closely associated with pancreatic islet dysfunction, enhanced inflammatory responses, and impaired vascular repair [43]. By inhibiting ODCs, eflornithine can effectively reduce abnormal polyamine levels, alleviate diabetes-related oxidative stress and inflammation, and improve wound healing and angiogenesis, suggesting that this gene and its inhibitors have important research value in the prevention and treatment of diabetes and its complications.

Importantly, integrating cellular heterogeneity analysis with metabolic pathway characterization and machine learning-based biomarker selection provides a potential framework for precision medicine in DFU. By identifying metabolically active keratinocyte subpopulations and their associated molecular signatures, this strategy enables patient stratification based on underlying biological mechanisms. Such an approach may facilitate the development of targeted metabolic interventions and personalized therapeutic strategies aimed at improving wound healing outcomes. This study used single-cell transcriptome analysis to identify the major cell types and subpopulations in DFU and controls. Key findings included the discovery that Kera1 cells exhibited high stemness and actively proliferated, capable of differentiating into Kera3 along a developmental trajectory. In DFU patients, Kera1 significantly enhanced CDH signaling interactions with fibroblasts and smooth muscle cells, implicated in the regulation of inflammation and proteolysis. Combined with differentially expressed genes and amino acid metabolism-related gene analysis, five key genes, RPL13, ODC1, RPL22L1, GATM, and GLUL, were identified using multiple machine learning approaches. ODC1, RPL22L1, and GLUL were significantly overexpressed in DFU patients and interacted with potential drugs, including eflornithine hydrochloride, highlighting their diagnostic and therapeutic value.

## CONCLUSION

This study demonstrates that amino acid metabolism dysregulation is closely associated with keratinocyte heterogeneity and may contribute to impaired wound healing in DFU. The Kera1 subset demonstrated stem-like characteristics, dynamic differentiation potential, and enhanced cadherin-mediated cell-cell communication, which may contribute to DFU pathogenesis. Functional analyses further linked Kera1 to stress response, proteolysis, and extracellular matrix reorganization processes likely involved in healing impairment. We also identified five amino acid metabolism-related genes (RPL13, ODC1, RPL22L1, GATM, and GLUL) as consistent diagnostic biomarkers for DFU. Notably, ODC1 showed promise as a therapeutic target, supported by molecular docking with eflornithine. These findings advance our understanding of keratinocyte subpopulations in DFU and propose potential diagnostic markers and targeted treatment approaches.

## LIMITATION

This study is primarily based on integrative bioinformatic and machine learning analyses of public transcriptomic datasets. Although these approaches provide systematic insights into amino acid metabolism-related alterations in DFU, they cannot establish causal relationships. Functional validation experiments are necessary to confirm the mechanistic roles of the identified pathways and candidate genes in keratinocyte dysfunction and impaired wound healing. Additionally, validation in larger, independent clinical cohorts will be necessary to further support their diagnostic and therapeutic potential.

## Figures and Tables

**Fig. (1):**
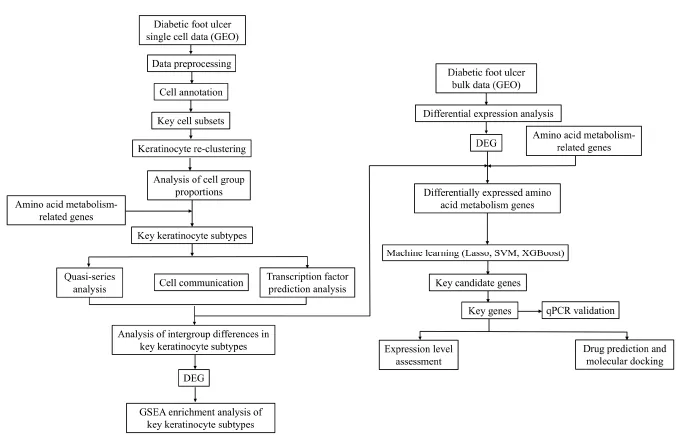
Flowchart of this study.

**Fig. (2):**
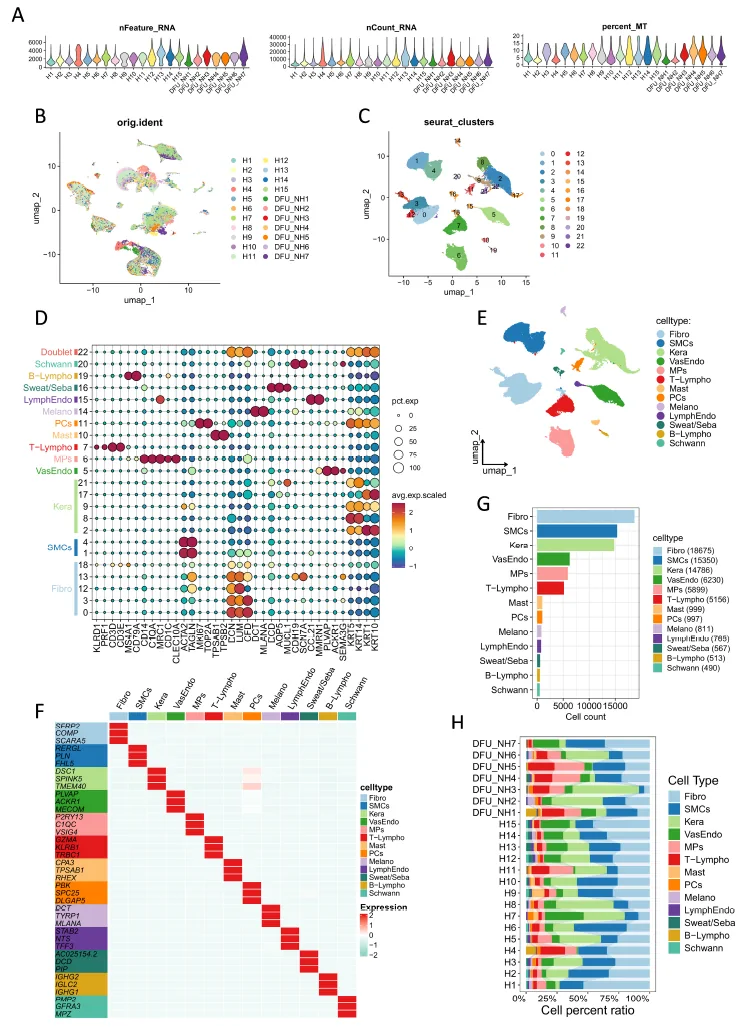
Single-cell transcriptome analysis and cell type annotation. (**A**) Violin plots displaying the nCount_RNA, nFeature_RNA, and percent.mt per cell after quality control. (**B**) UMAP projection of all cells before batch effect correction. (**C**) UMAP plot showing the 23 unsupervised clusters identified in the single-cell dataset before annotation. (**D**) Dot plot of canonical marker gene expression used for cell type annotation. Dot size represents the percentage of cells expressing the gene, and color intensity represents the average expression level. (**E**) UMAP plot of all cells after annotation, colored by the 13 identified cell types. (**F**) Heatmap of the top 3 marker genes for each cell cluster, confirming cluster identity. (**G**) Bar plot showing the absolute cell numbers for each cell type in the Healthy (**H**) and Diabetic Foot Ulcer Non-Healing (DFU_NH) groups. (H) Stacked bar plot showing the relative proportion (percentage) of each cell type across all samples in the H and DFU_NH groups.

**Fig. (3):**
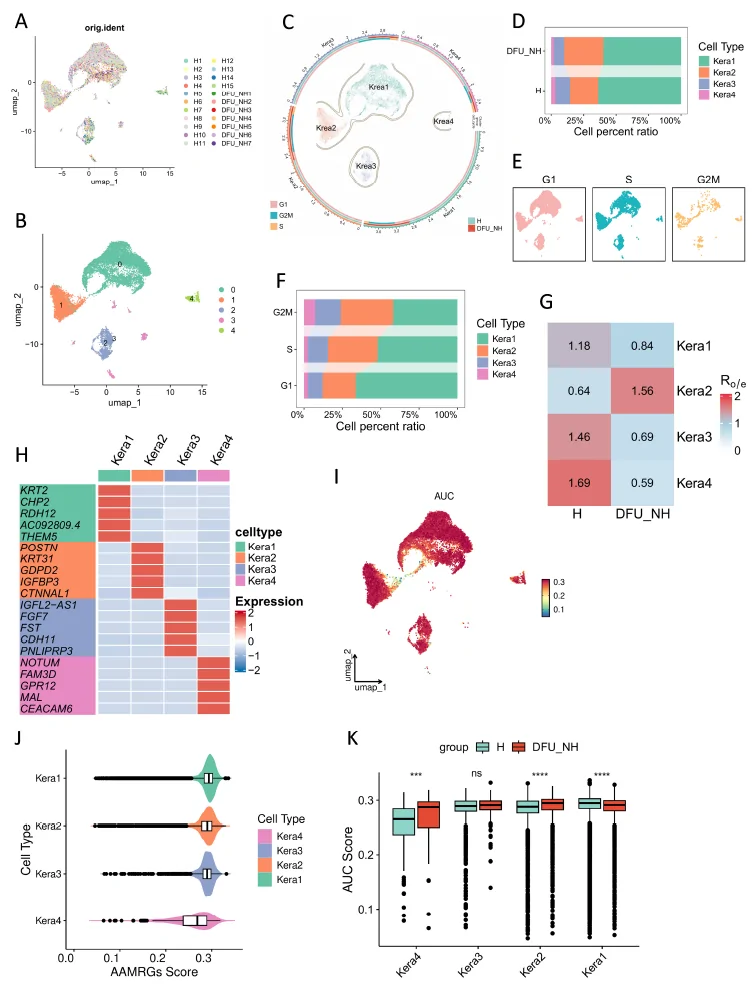
Identification and functional characterization of keratinocyte subsets. (**A**) UMAP projection of keratinocytes after batch effect correction. (**B**) UMAP plot showing the 5 initial clusters identified from keratinocytes. (**C**) UMAP plot defining the four final keratinocyte subtypes: Kera1, Kera2, Kera3, and Kera4. (**D**) Stacked bar plot showing the proportion of each keratinocyte subtype in the H and DFU_NH groups. (**E**) UMAP plot colored by cell cycle phase (G1, S, G2M), showing the cell cycle distribution of keratinocytes. (**F**) Stacked bar plot showing the proportion of each keratinocyte subtype in different cell cycle phases. (**G**) Ratio of observed to expected (Ro/e) scores for each keratinocyte subtype in H and DFU_NH groups, indicating functional enrichment. (**H**) Heatmap of the top 5 differentially expressed marker genes for each keratinocyte subtype. (**I**) UMAP plot colored by the AUCell score, representing the activity of the amino acid metabolism-related gene set. (**J**) Violin plots comparing the distribution of AUCell scores across the four keratinocyte subtypes. (**K**) Violin plots comparing the AUCell scores of the Kera1 subset between the H and DFU_NH groups.

**Fig. (4):**
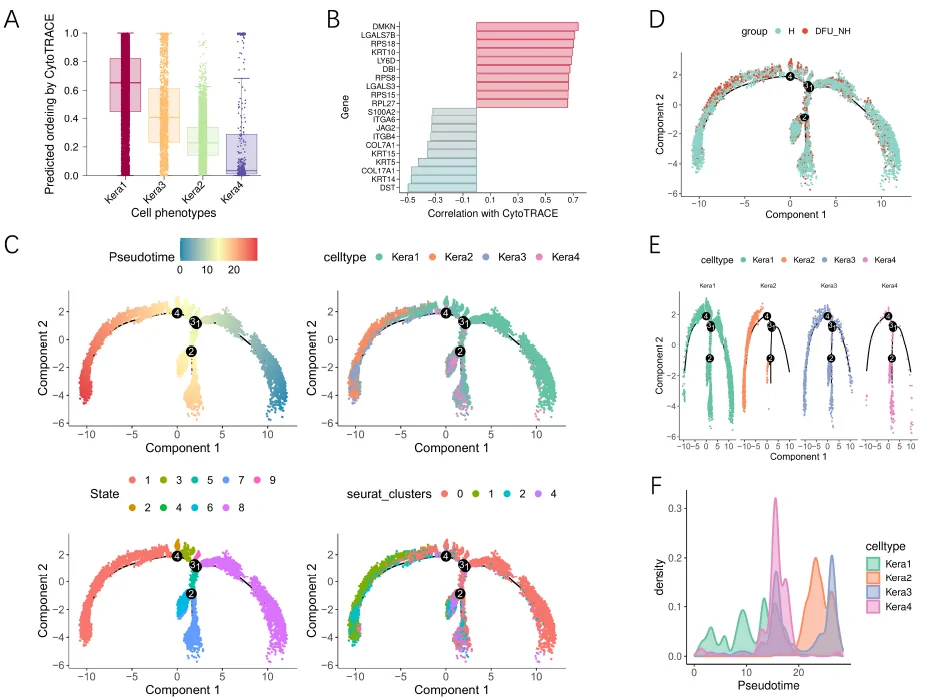
Kera1 Displays Stemness and Initiates Differentiation Toward Kera3. (**A**) Box plot of stemness scores for each keratinocyte subtype predicted by the CytoTRACE algorithm. A higher score indicates greater stemness and lower differentiation. (**B**) Expression patterns of representative genes that show the strongest positive and negative correlation with CytoTRACE scores across keratinocyte subtypes. (**C**) Pseudotime trajectory analysis of keratinocyte subtypes using Monocle. Each dot represents a cell and is colored by its subtype. (**D**) Distribution of cells along the pseudotime trajectory, colored by the sample group (H or DFU_NH). (**E**) Proportion of cells from H and DFU_NH groups at different stages of the pseudotime trajectory. (**F**) Smoothed density distribution of cells from Kera1, Kera2, Kera3, and Kera4 groups along the pseudotime trajectory, highlighting the differential abundance of cells at various differentiation stages.

**Fig. (5):**
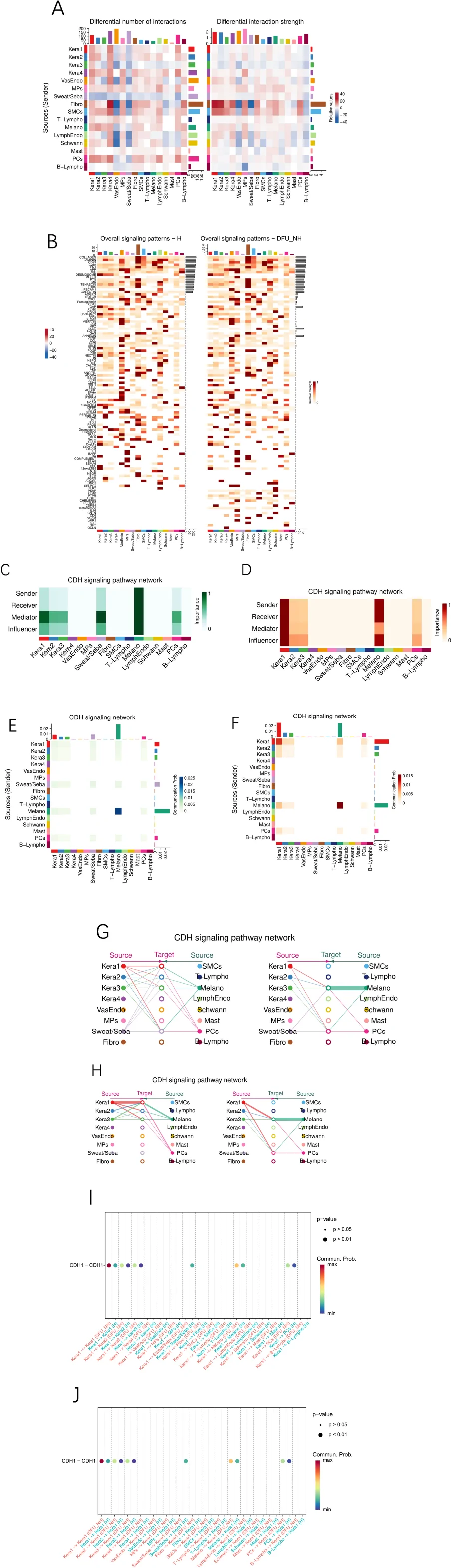
Enhanced CDH signaling highlights enhanced Kera1 interaction in DFU_NH. (**A**) Heatmap of differences in communication between cell types. (**B**) Heatmap of changes in overall signaling pathway activity between the H and DFU_NH groups. (**C**) Heatmap of the role of CDH signaling in the H group. (**D**) Heatmap of the role of CDH signaling in the DFU_NH group. (**E**) Heatmap of CDH signaling communication probability in the H group. (**F**) Heatmap of CDH signaling communication probability in the DFU_NH group. (**G**) CDH signaling hierarchy diagram in the H group. (**H**) CDH signaling hierarchy diagram in the DFU_NH group. (**I**) Expression intensity of ligand-receptor pairs under CDH signaling (Kera1 as the sender). (**J**) Expression intensity of ligand-receptor pairs under CDH signaling (Kera1 as the receiver). Dot size and color intensity represent the computed communication probability.

**Fig. (6):**
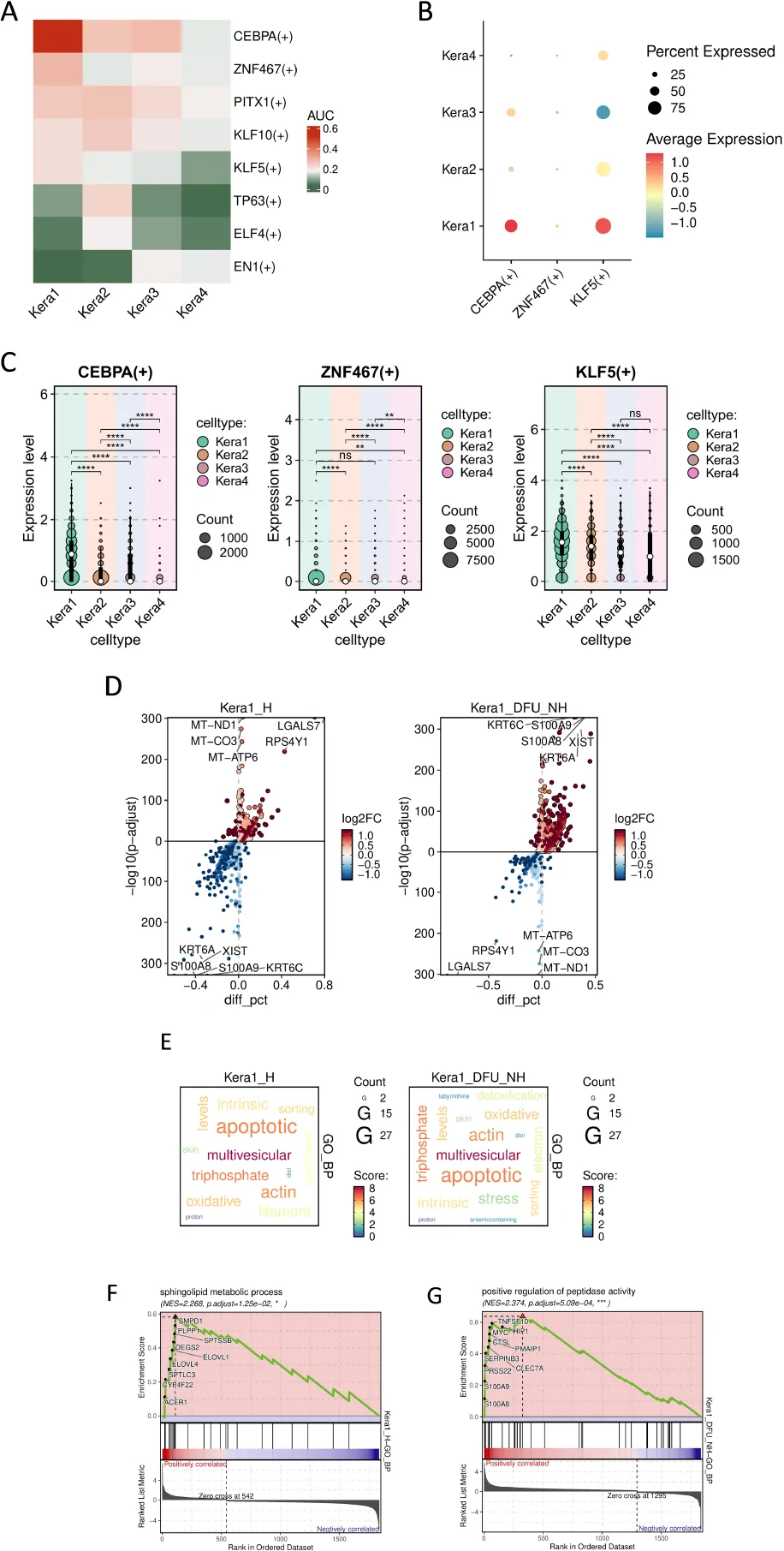
Key transcription factors and functional enrichment of Kera1 in DFU. (**A**) Heatmap of the cell and the top 5 transcription factor activity. (**B**) Dot plot visualizing the expression and regulatory activity of the key TFs CEBPA, ZNF467, and KLF5 across keratinocyte subtypes. (**C**) Boxplot of key transcription factor expression. (**D**) Volcano plots of differentially expressed genes (DEGs) in the Kera1 subset when comparing the DFU_NH group to the H group. Genes with significant up-regulation are shown in red, and down-regulation in blue. (**E**) Word cloud generated from the Gene Ontology Biological Process (GO-BP) enrichment analysis of DEGs in Kera1, where the size of the term corresponds to its statistical significance. (**F, G**) Gene Set Enrichment Analysis (GSEA) plots for the Kera1 subset in the H (**F**) and DFU_NH (**G**) groups, showing representative enriched biological processes. The green curve depicts the enrichment score, and the ranked list metric reflects the correlation of genes with the H or DFU_NH phenotype.

**Fig. (7):**
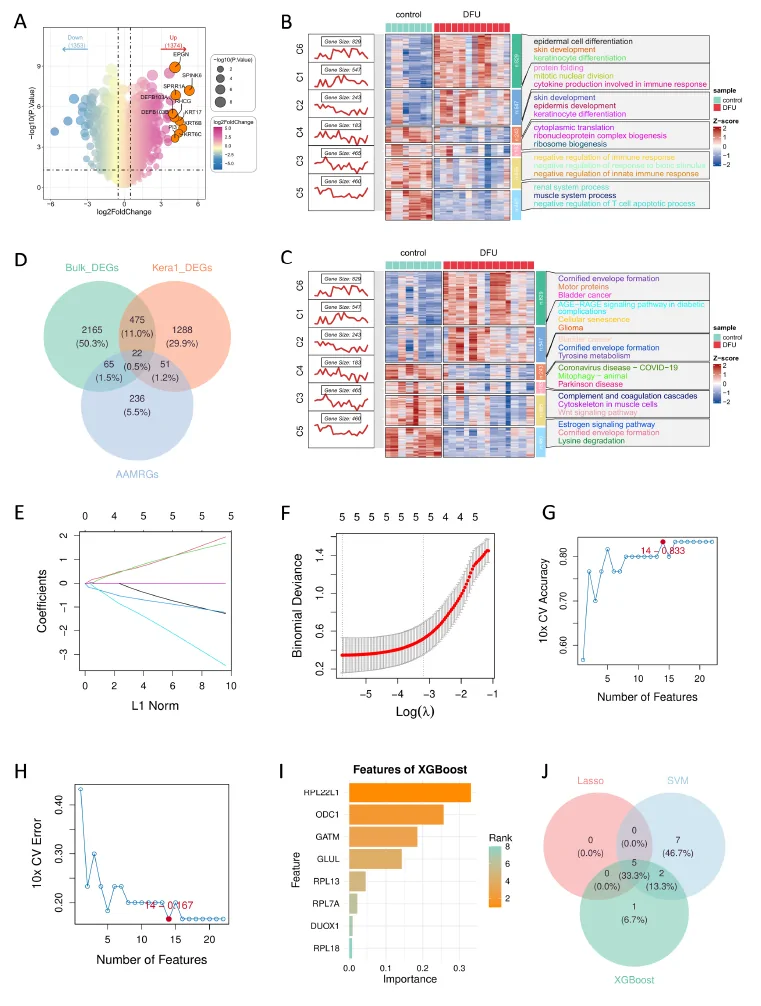
Screening of candidate genes related to amino acid metabolism. (**A**) Identification of differentially expressed genes. Significantly up- regulated genes are in red, down-regulated in blue. The top 10 up-regulated genes are labeled. (**B**) GOBP enrichment analysis of differentially expressed genes. (**C**) Kyoto Encyclopedia of Genes and Genomes (KEGG) enrichment analysis of differentially expressed genes. (**D**) Identification of disease-related amino acid metabolism genes. (**E**) Lasso regression path diagram. Each curve represents a gene. (**F**) Lasso regression cross-validation curve. (**G-H**) SVM algorithm. (**I**) Bar chart of candidate key gene importance ranking. (**J**) Intersection genes of three machine learning algorithms. Venn diagram showing the intersection of the key candidate genes identified by the three machine learning algorithms (Lasso, SVM, XGBoost), yielding the final five key genes.

**Fig. (8):**
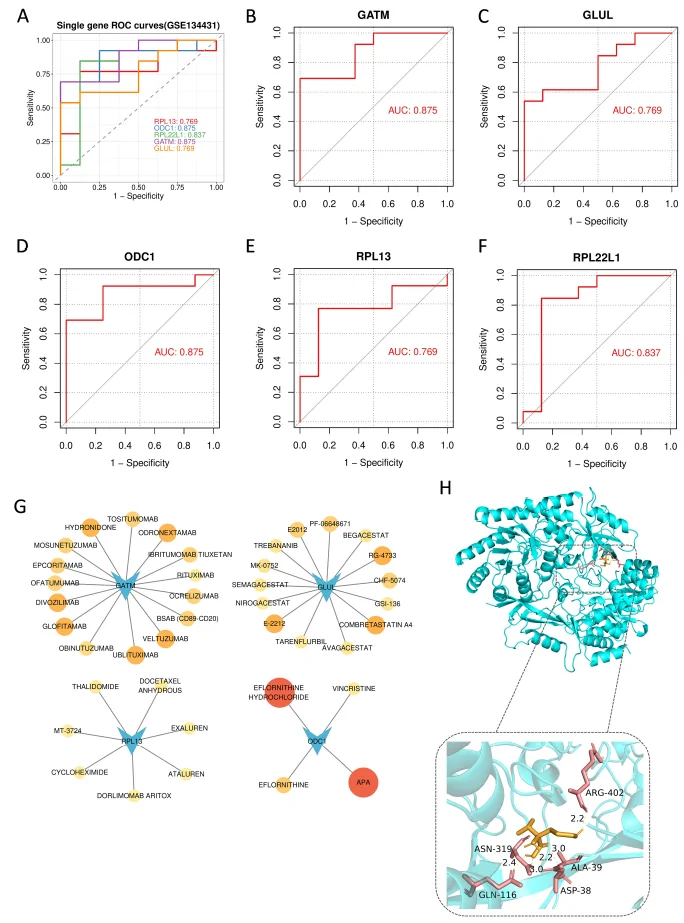
Diagnostic performance of key genes and identification of potential therapeutic targets in DFU. (**A-F**) Receiver Operating Characteristic (ROC) curves evaluating the diagnostic performance of the five key genes (RPL13, ODC1, RPL22L1, GATM, GLUL) in the training dataset GSE134431. The Area Under the Curve (AUC) value for each gene is displayed on the plot. (**G**) Drug-gene interaction network. Nodes represent the five key genes and predicted interacting drugs. Edges connect genes to their potential therapeutic compounds. (**H**) Molecular docking.

**Fig. (9):**
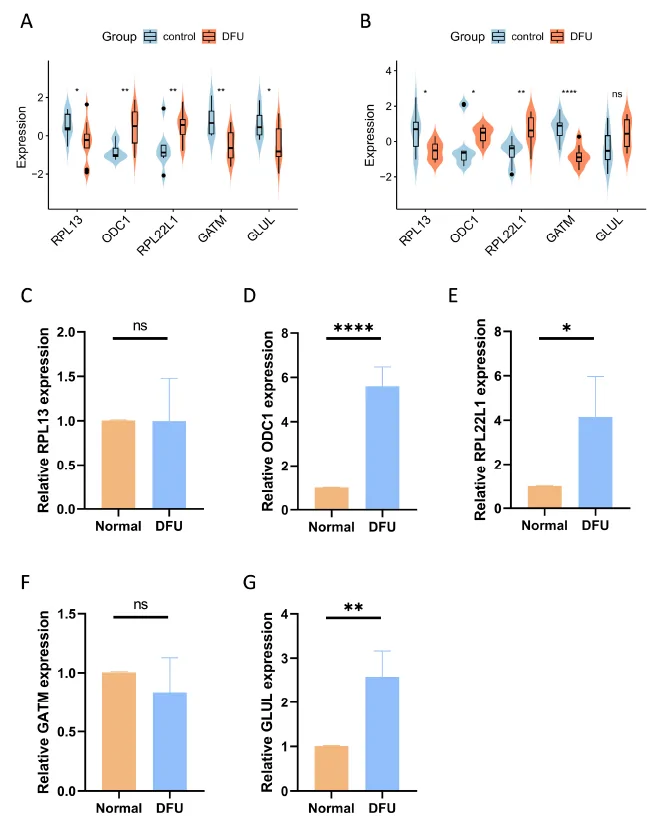
Differential expression of key genes in DFU patients and controls. (**A, B**) Violin plots showing the expression distribution of the five key genes in the bulk RNA-seq training set GSE134431 (**A**), and validation set GSE199939 (**B**). (**C-G**) qPCR validation of the expression levels of RPL13 (**C**), ODC1 (D), RPL22L1 (**E**), GATM (**F**), and GLUL (**G**) in clinical tissue samples from DFU patients and healthy controls. Gene expression levels are presented as relative quantification (RQ). ns: not significant, *P < 0.05, **P < 0.01, ****P < 0.0001.

**Table 1:** Information on the datasets utilized in this research.

Dataset	Sample (Controls)	Sample (Patients)	Sequencing Platform
GSE199939	11	10	GPL24676
GSE134431	8	13	GPL18573
GSE7014	6	20	GPL570

**Table 2:** The binding energy of key genes and their potential drugs.

Gene	Drug	Binding Energy (kcal/mol)
ODC1	Eflornithine	-5.2

## Data Availability

All the RNA-sequencing data and single-cell sequencing data of DFU patients were acquired from the Gene Expression Omnibus database (GEO, https://www.ncbi.nlm.nih.gov/geo/).

## LIST OF ABBREVIATIONS

AAMRGs: Amino Acid Metabolism-Related Genes
AUC: Area Under the Curve
AUCell: Area Under the Curve for Cells
BP: Biological Process
CEBPA: CCAAT Enhancer Binding Protein Alpha
DEGs: Differentially Expressed Genes
DFU: Diabetic Foot Ulcer
DFU_NH: Diabetic Foot Ulcer Non-Healing
DGIdb: Drug-Gene Interaction database
FC: Fold Change
FGFs: Fibroblast Growth Factors
Fibro: Fibroblasts
GATM: Glycine Amidinotransferase
GEO: Gene Expression Omnibus
GLUL: Glutamate-Ammonia Ligase
GO: Gene Ontology
H: Healthy Control
KEGG: Kyoto Encyclopedia of Genes and Genomes
Kera: Keratinocytes
KLF5: Krüppel-Like Factor 5
Lasso: Least Absolute Shrinkage and Selection Operator
MPs: Macrophages
MSigDB: Molecular Signatures Database
mTOR: Mechanistic Target of Rapamycin
ODC1: Ornithine Decarboxylase 1
PCA: Principal Component Analysis
PDB: Protein Data Bank
qPCR: Quantitative Polymerase Chain Reaction
RAGE: Receptor for Advanced Glycation End products
ROC: Receiver Operating Characteristic
RPL13: Ribosomal Protein L13
RPL22L1: Ribosomal Protein L22 Like 1
SASP: Senescence-Associated Secretory Phenotype
SCENIC: Single-Cell Regulatory Network Inference and Clustering
scRNA-seq: Single-cell RNA sequencing
SMCs: Smooth Muscle Cells
SVM: Support Vector Machine
T-Lympho: T Lymphocytes
TF: Transcription Factor
t-SNE: t-distributed Stochastic Neighbor Embedding
UMAP: Uniform Manifold Approximation and Projection
VasEndo: Vascular Endothelial Cells
XGBoost: Extreme Gradient Boosting

## STANDARDS OF REPORTING

STROBE guidelines and methodologies were followed.

## SUPPLEMENTARY MATERIAL

Supplementary material is available on the publisher’s website along with the published article.
**Supplementary Figure 1** **.** (A) Hypervariable genes. (B) Elbow plot. (C) Cluster tree of cell populations. (D) Re-clustered hypervariable genes. (E) Re-clustered elbow plot. (F) Re-clustered cluster tree.
**Supplementary Figure 2** **.** (A-F) ROC curves of individual genes in the validation set GSE199939. (G-L) ROC curves of individual genes in the validation set GSE7014.
