# Supplementary material for: Single-cell Sequencing and Machine Learning Identify Amino Acid Metabolism-related Biomarkers and Regulatory Mechanisms in Diabetic Foot Ulcers
Source: Endocr Metab Immune Disord Drug Targets. 2026 May 21;26:E18715303451779. doi: 10.2174/0118715303451779260511203500 (PMC13635959; doi:10.2174/0118715303451779260511203500)
Supplement: Supplementary file 1 [file EMIDDT-26-E18715303451779_SD1.pdf]

Supplementary Material

Single-cell Sequencing and Machine Learning Identify Amino Acid Metabolism-related Biomarkers and Regulatory Mechanisms in Diabetic Foot Ulcers

Wenting Wang[1,*], Zhengguo Xia[1], Yin Wang[1], Fan Wang[1] and Feng Han[1]

[1]Wound Repair & Plastic and Anesthetic Surgery, the First Affiliated Hospital of Anhui Medical University, North District, Hefei City, Anhui Province 230022, China

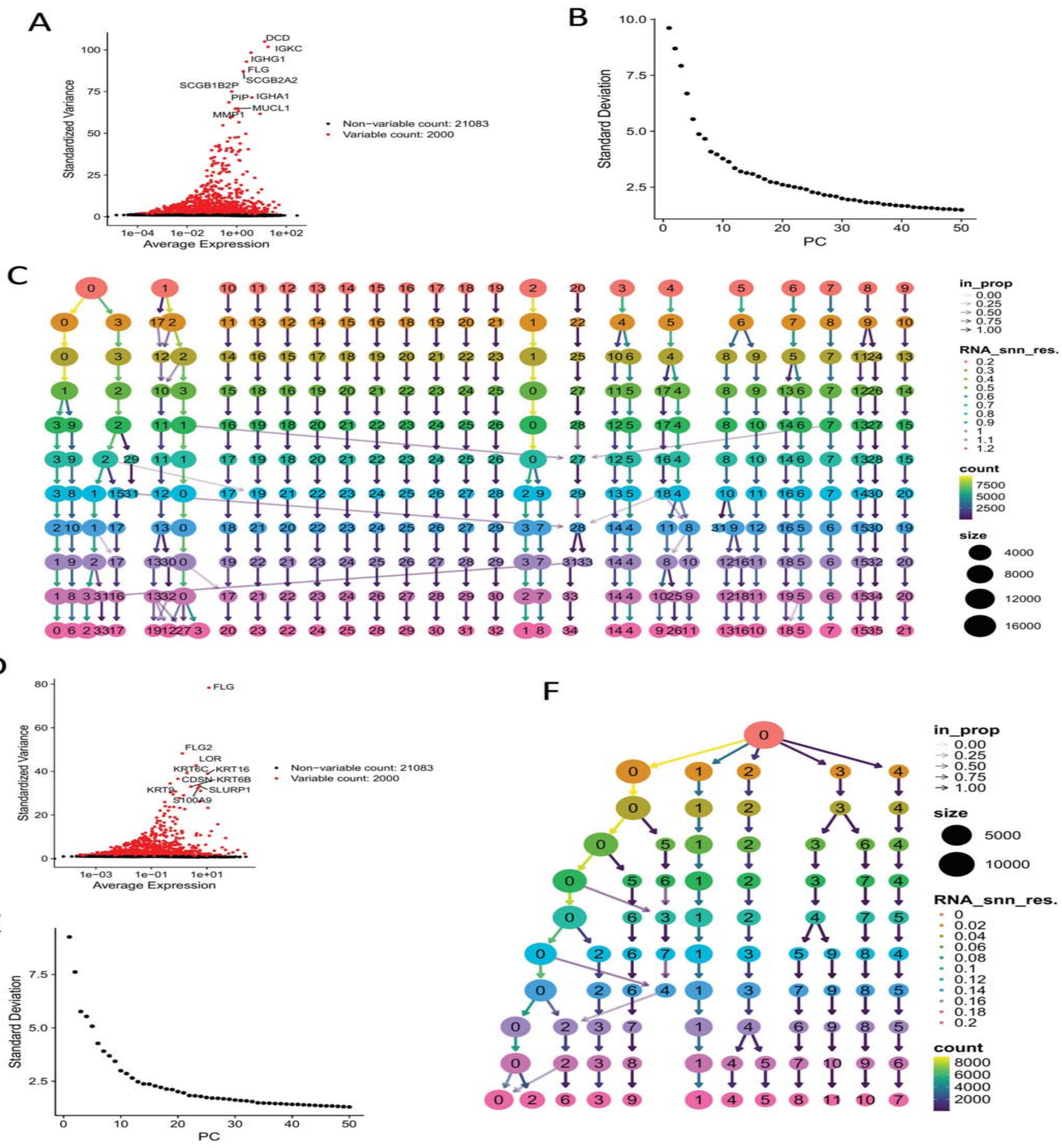

Fig. (S1).

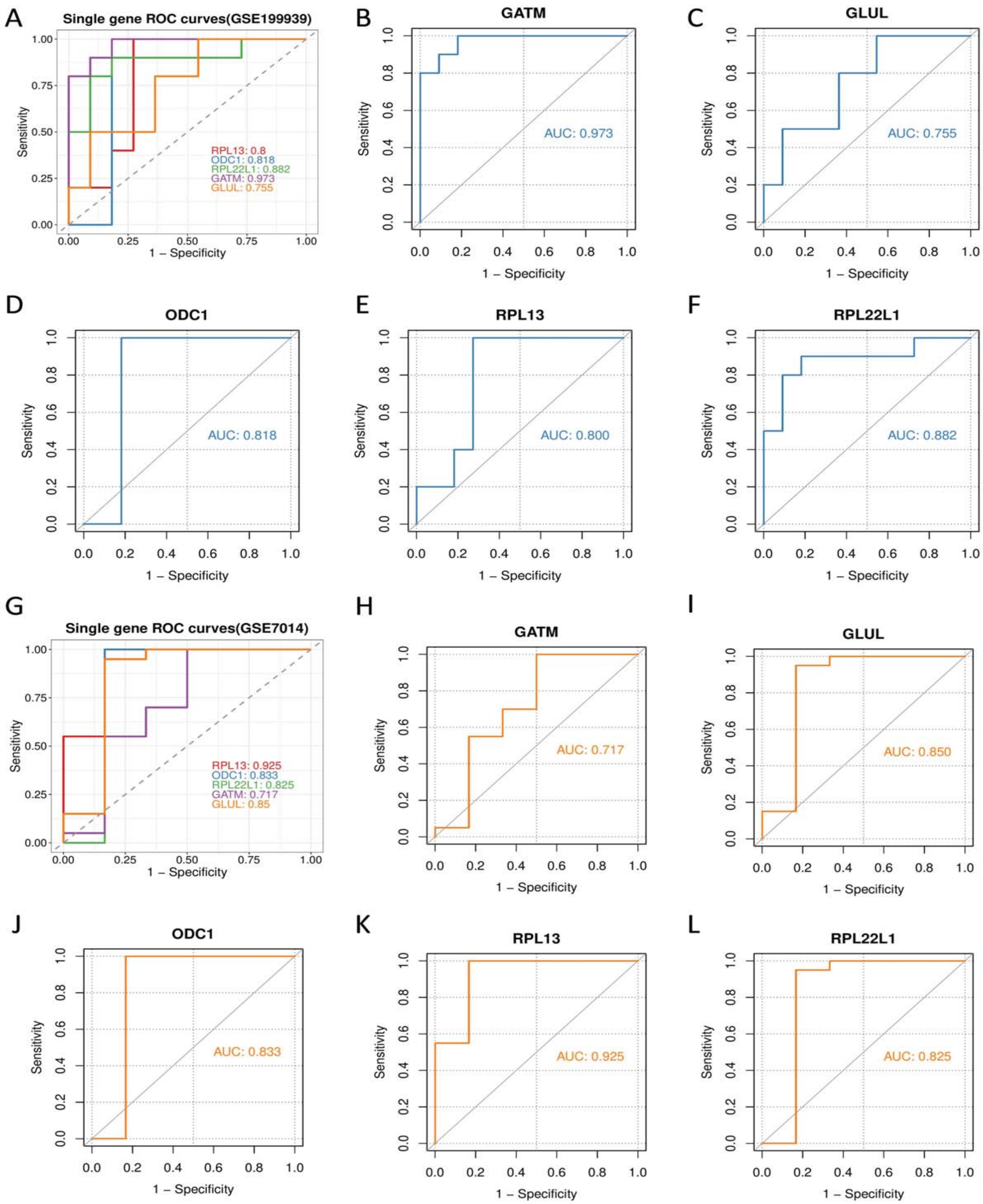
A
Single gene ROC curves(GSE199939)
RPL13: 0.8
ODC1: 0.818
RPL22L1: 0.882
GATM: 0.973
GLUL: 0.755
Sensitivity
1 – Specificity
B
GATM
AUC: 0.973
C
GLUL
AUC: 0.755
D
ODC1
AUC: 0.818
E
RPL13
AUC: 0.800
F
RPL22L1
AUC: 0.882
G
Single gene ROC curves(GSE7014)
RPL13: 0.925
ODC1: 0.833
RPL22L1: 0.825
GATM: 0.717
GLUL: 0.85
H
GATM
AUC: 0.717
I
GLUL
AUC: 0.850
J
ODC1
AUC: 0.833
K
RPL13
AUC: 0.925
L
RPL22L1
AUC: 0.825

Fig. (S2)
